# Genetic Diversity and Population Structure in Captive Populations of Formosan Sambar Deer (*Rusa unicolor swinhoei*)

**DOI:** 10.3390/ani13193106

**Published:** 2023-10-05

**Authors:** Hsiao-Mei Liang, Kuo-Tai Yang, Yu-Tzu Cheng, Shen-Chang Chang, Cheng-Yung Lin, Ming-Yang Tsai, Der-Yuh Lin, Kuo-Hsiang Hung

**Affiliations:** 1 Southern Region Branch, Livestock Research Institute, Ministry of Agriculture, Pingtung 912013, Taiwan; hlliang@mail.tlri.gov.tw (H.-M.L.); macawh@mail.tlri.gov.tw (S.-C.C.); 2 Department of Animal Science, National Pingtung University of Science and Technology, Pingtung 912301, Taiwan; ktyang@mail.npust.edu.tw; 3 Department of Forestry, Pingtung University of Science and Technology, Pingtung 912301, Taiwan; yvcheneng0408@gmail.com; 4 Livestock Management Division, Livestock Research Institute, Ministry of Agriculture, Tainan 71246, Taiwan; jengyong@mail.tlri.gov.tw (C.-Y.L.); mytsai@mail.tlri.gov.tw (M.-Y.T.); 5 Genetics and Physiology Division, Livestock Research Institute, Ministry of Agriculture, Tainan 71246, Taiwan; lin0429@mail.tlri.gov.tw; 6 Graduate Institute of Bioresources, National Pingtung University of Science and Technology, Pingtung 912301, Taiwan

**Keywords:** Formosan sambar deer, inbreeding, microsatellite, parentage analysis, genetic diversity

## Abstract

**Simple Summary:**

*Rusa unicolor swinhoei* is an economically important animal in Taiwan, owing to the market demand for its velvet antler. In this study, we investigated the genetic diversity and structure of Formosan sambar deer using microsatellites. The observed genetic diversity was low, which is likely attributable to inbreeding and bottleneck effects. Moreover, this study revealed two distinct genetic groups within the captive populations and found no significant population genetic structure among the captive populations in Taiwan. These findings have the potential to improve breeding management and contribute to the mitigation of inbreeding, thereby promoting the productive potential of Formosan sambar deer.

**Abstract:**

Formosan sambar deer (*Rusa unicolor swinhoei*) are of great economic significance in Taiwan, resulting in a substantial increase in deer farming to meet the high demand for velvet antlers. Inbreeding depression and reduced genetic variability can lead to the deterioration of captive populations. In this study, 239 Formosan sambar deer were genotyped using 13 microsatellites to analyze their genetic diversity and population genetic structure. Our results indicate a high-resolution power of these microsatellites in individual discrimination and parentage analysis. However, captive populations exhibit a low level of genetic diversity, likely because of inbreeding and bottleneck effects. Both principal coordinate analysis (PCoA) and STRUCTURE analyses revealed two distinct and segregated genetic groups within the captive populations and indicated no clear population genetic structure among the captive populations. Introducing new genetic material from the wild through translocation offers a potential solution for mitigating the impact of inbreeding and enhancing genetic diversity. The comprehensive information obtained from these genetic analyses is crucial for the development of effective breeding strategies aimed at preserving and enhancing Formosan sambar deer populations.

## 1. Introduction

*Rusa unicolor* is a large ungulate distributed throughout Southeast and South Asia, including South China, India, Nepal, Cambodia, Vietnam, Taiwan, and Malaysia [1,2]. Formosan sambar deer (*R. unicolor swinhoii*) is a subspecies endemic to Taiwan that is categorized as a protected species in Taiwan [3]. A significant decline in the number and geographic distribution of wild populations of Formosan sambar deer in Taiwan was caused by habitat loss and hunting during the 20th century [3,4]. Formosan sambar deer have become an important economic animal in Taiwan since 1963; a large number of deer are kept in farms due to the increasing demands for velvet antlers [5]. Velvet antler has been used in traditional Chinese medicine and as a health food for over 2000 years, owing to its benefits to human health [6,7]. They contain multiple chemical substances such as peptides, amino acids, phospholipids, sterols, lipoproteins, carbohydrates, and inorganic substances, which are beneficial for human health [8]. Pharmacological studies have indicated that these active compounds have inhibitory effects on arthritis [9], immunomodulatory activities [10], and anti-narcotic effects [11].

Maintaining genetic diversity is a critical aspect of economic animal populations, and genetic variation within these animals plays a pivotal role in ensuring their adaptability to changing environments [12]. Inbreeding reduces heterozygosity and increases homozygosity, leading to a decrease in the overall genetic variation within a population. The loss of genetic variations may lead to reduced fitness and increased susceptibility to genetic disorders [13]. Furthermore, inbred populations are more susceptible to the effects of genetic drift and exhibit a reduced adaptive potential, which limits their ability to respond to environmental changes [14]. For captive populations, the main challenges include small population size, genetic degeneration with a consequent loss of genetic variation, and inbreeding depression [15]. Understanding the consequences of inbreeding is crucial for managing captive populations and maintaining their long-term viability.

Microsatellites, also known as simple sequence repeats (SSRs), consist of simple tandem sequence repeats of 1–6 to nucleotide units and are distributed throughout the genome [16]. Owing to their extensive polymorphisms, they have been extensively utilized to study genetic diversity, genetic differentiation among populations, and population structures [17]. Microsatellites are suitable for analyzing both parentage and inbreeding levels [18,19]. Numerous studies have utilized microsatellites to assess the genetic diversity, population structure, and parentage of captive animals, including Chinese water deer [19], sika deer [20], Asian woolly-necked storks [21], and white-tailed deer [22]. Historically, studies on the Formosan sambar deer have primarily focused on wild populations, including habitat selection [3] and bark-stripping behavior [23]. However, Li et al. [24] employed mitochondrial DNA to investigate the phylogeography of wild populations and indicated the glacial refugium in the northern area of Taiwan’s Central Mountain Range.

The number of captive deer in Taiwan is relatively small when compared with that in regions such as Europe, America, New Zealand, and China. When a captive population is too small, there is a higher risk of increased inbreeding within the population. This can result in the reduced expression of traits related to population survival and reduced genetic diversity. Therefore, breeding systems for captive deer are crucial in Taiwan. The objectives of this study were as follows:

(1) To assess whether the microsatellites utilized in this study are suitable for individual discrimination and parentage analysis of the Formosan sambar deer.
(2) To examine the genetic variation and structure of captive deer farms in Taiwan.

The findings of this study will be valuable for designing appropriate management and breeding strategies for captive Formosan sambar deer in Taiwan, ultimately aiding the conservation and sustainable utilization of this species.

## 2. Materials and Methods

### 2.1. Sample Collection, and DNA Extraction

Blood samples were collected from 239 individuals, representing 15 captive populations of Formosan sambar deer in Taiwan (Table 1). Samples were captured under a license with permission granted by Southern Region Branch, Livestock Research Institute, Ministry of Agriculture, Taiwan (permit number 104-10). After cutting the velvet antler of the Formosan sambar deer, blood samples were collected in anticoagulant ACD tubes. Genomic DNA was extracted from the whole blood samples using the phenol/chloroform method [25].

### 2.2. Microsatellite Analysis

In this study, a set of 13 microsatellite was employed for individual genotyping of Formosan sambar deer [26,27,28,29] (Table 2). Polymerase chain reaction (PCR) amplification was performed in a 25 μL reaction, with the forward primers labelled with fluorescent dye. The PCR amplification program consisted of denaturation at 95 °C for 3 min, followed by 30 cycles at 92 °C for 30 s, primer-specific annealing temperatures for 30 s, extension at 72 °C for 30 s, and a final extension at 72 °C for 10 min. The PCR products were preserved at 4 °C. The PCR products were subjected to capillary electrophoresis on an ABI 3730 automated sequencer, and allelic sizes were determined using the Gene Mapper software (version 4.0; Applied Biosystems, Waltham, MA, USA).

### 2.3. Discrimination Ability of the Microsatellites

The power of discrimination (PD) was assessed for each microsatellite: PD = 1 − ΣPi^2^, where: Pi is the frequency of genotype i [30]. We computed the cumulative power of discrimination (cPD) for the 13 microsatellites using the formula: cPD = 1 − [(1 − PD_1_) (1 − PD_2_) … (1 − PD_13_)] [31]. Additionally, we assessed the probabilities of identity of unrelated individuals (PI) and siblings (PIsibs) using Cervus v3.0.7. These probabilities represent the likelihood of two randomly selected unrelated individuals and siblings within a population of the same genotype [32]. The order of discrimination power and optimal combination of microsatellites were estimated from the PD values [30].

### 2.4. Genetic Diversity and Differentiation Analysis

Genetic diversity parameters, including alleles per locus (A), observed heterozygosity (Ho), and expected heterozygosity (He) were evaluated for each locus and population using the GenAlEx 6.5 software [33]. Additionally, we examined the inbreeding coefficient (F_IS_), which represents the level of inbreeding within a population, and genetic differentiation (Fst) and Nei’s genetic distance between populations. PowerMarker v3.25 was used to assess the polymorphic information content (PIC) for each marker [34]. The Nei’s genetic distance of pairwise population matrices were then used to construct neighbor-joining tree using MEGA software v11 [35]. Statistical analyses were performed to test whether the captive population of Formosan sambar deer had experienced a genetic bottleneck. We utilized the Bottleneck program [36,37] to examine departures from the drift-mutation equilibrium. A transient excess of expected heterozygosity relative to that expected under the mutation-drift equilibrium may indicate a bottleneck. Statistical analysis was performed using the Wilcoxon test, considering three mutation models: the stepwise mutation model (SMM) [38], infinite allele model (IAM) [39], and two-phase model (TPM) [40] in Bottleneck program. Isolation by distance (IBD) was analyzed by regressing pairwise population estimates of Nei’s genetic distances on the geographical distance (km) between all pairs of sample location. Mantel’s test was implemented with 9,999 permutation GenAlEx 6.5 software [33].

### 2.5. Assessment of Kin Relationships

ML-RELATE calculates relatedness and relationships using maximum-likelihood estimation [41]. Maximum likelihood tests were employed to estimate the most probable relationships between pairs of individuals, including unrelated (U), full-sibling (FS), half-sibling (HS), and parent-offspring (PO) relationships [41].

### 2.6. Population Structure Analysis

Principal coordinate analysis (PCoA) is a multivariate statistical technique used to visualize and explore the similarities or dissimilarities between samples based on a distance or dissimilarity matrix. In this study, PCoA was performed using the GenAlex 6.5 software to identify and illustrate the genetic clusters within the dataset [33]. In addition to PCoA, we employed a model-based Bayesian clustering approach using STRUCTURE version 2.3.4 to analyze allele frequencies at each locus and infer the population genetic structure [42]. For each K, this analysis was run for 1,000,000 length of the burn-in period of the Markov chain Monte Carlo (MCMC) with 10 iterations, which used the correlated allelic frequencies under the admixture model. We employed the web version of the Structure Harvester software (https://taylor0.biology.ucla.edu/structureHarvester/) (accessed on 10 July 2023) to estimate the ΔK (delta K) values for each genetic group and infer the optimal number of K (genetic clusters) [43].

## 3. Results

### 3.1. Discrimination Ability of 13 Polymorphic Microsatellite Markers

In the present study, a total of 83 alleles were identified among 239 Formosan sambar deer screened through 13 microsatellite loci, and all loci exhibited polymorphisms. The average discrimination power (PD) was 0.421 and ranged from 0.0041–0.7850. Ca18 and Ca71 exhibited the highest and lowest discrimination powers, respectively. The probabilities of identity for unrelated individuals (PI) and siblings (PIsibs) were evaluated for each locus and ranged from 0.073 to 0.992 and 0.375 to 0.991, respectively. Ca18 and Ca71 showed the lowest and highest values for PI and PIsibs, respectively (Table 3).

**Figure 1:**
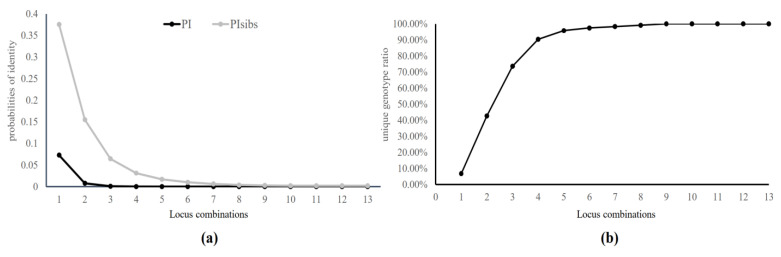
The discrimination power in locus combination. (**a**) Probability of identity of unrelated individuals (PI) and siblings (PIsibs); (**b**) unique genotype ratio.

Additionally, we calculated the cumulative PI and PIsibs values using optimal combinations of all 13 microsatellite loci. The cumulative PI and PIsibs values were 4.589 × 10^−7^ and 1.871 × 10^−3^, respectively (Figure 1a). The Ca18 locus exhibited the highest discriminatory power. In contrast, the Ca71 locus had the lowest discriminatory power (Table 3). When focusing solely on the Ca18 locus, 223 individuals shared the same genotype, and were therefore indistinguishable from each other (unique genotype ratio = 6.695%). However, nine polymorphic microsatellite loci, including Ca18, Ca13, Ca67, RT1, TGLA, Ca30, BM4107, CEH-5, and Cu10, allowed the discrimination of all individuals within the captive populations (unique genotype ratio = 100%, Figure 1b).

### 3.2. Genetic Diversity of Formosan Sambar Deer

For all samples, the allele number (A) ranged from 2.000 (Cu02, Ca30, and Ca71) to 15.000 (Ca18), with a mean value of 6.385 alleles. The polymorphism information content (PIC) values ranged from 0.004 (Ca71) to 0.759 (Ca18), with a mean value of 0.388 (Table 3). Of the 13 sets of microsatellites, 4 sets displayed PIC < 0.25, 4 sets exhibited 0.25 < PIC < 0.50, and the remaining exhibited PIC > 0.5.

The observed heterozygosity (Ho) and expected heterozygosity (He) ranged from 0.004 (Ca71) to 0.706 (Ca67) and 0.004 (Ca71) to 0.785 (Ca18), respectively. The mean Ho and He values were 0.299 and 0.421, respectively. The inbreeding coefficient (F_IS_) ranged from to −0.002 (Ca71) to 0.983 (Ca30). These results indicated that there was a heterozygote deficiency observed at all loci, except for loci Cu02, Cu09, Cu10, and Ca71 (Table 3). All microsatellite loci showed significant deviations from HWE (*p* < 0.05), except for Cu02, Cu09, and Ca71.

There was a significant excess of heterozygosity in the entire captive population of Taiwan relative to the mutation-drift expectation under both SMM (*p* = 0.001) and TPM (*p* = 0.008), but not under IAM (*p* = 0.685). Furthermore, this test conducted on captive populations of Formosan sambar deer revealed evidence of a bottleneck.

### 3.3. Genetic Variations and Population Genetic Structure in 15 Captive Populations

The number of alleles (A) varied from 2.231 (NTO and YUN2) to 3.923 (TNN4 and TNN5) in 15 captive populations. The observed heterozygosity (Ho) and expected heterozygosity (He) ranged from 0.222 to 0.346 and 0.299 to 0.449, respectively, with mean values of 0.291 and 0.375, respectively (Table 4). Populations TNN1 and TNN2 exhibited the lowest and highest Ho values, whereas populations NTO and KHH3 displayed the lowest and highest He values, respectively. The analysis revealed that all 15 captive populations of Formosan sambar deer displayed heterozygosity deficiency, as indicated by the positive values of the inbreeding coefficient (F_IS_) (Table 4). These findings indicate that inbreeding occurs within captive populations, where individuals are more likely to mate with close relatives.

**Figure 2:**
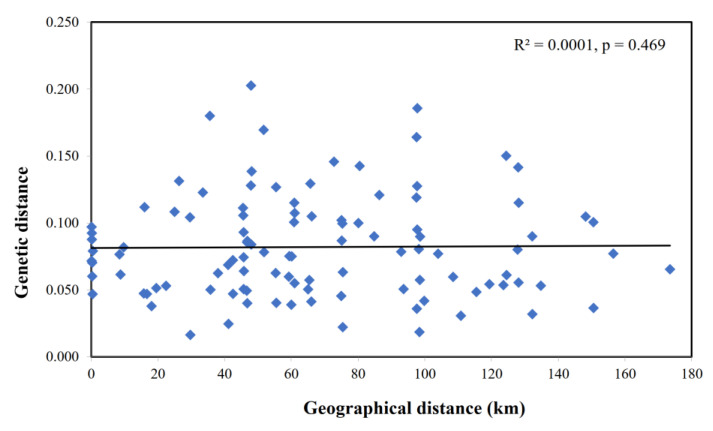
Geographical distance versus Nei’s genetic distance for captive populations of *Rusa unicolor swinhoei*. Correlations and probabilities were estimated from a Mantel test with 9999 permutation.

We also conducted a pairwise population Fst analysis for the 15 captive populations of Formosan sambar deer, and the calculated values ranged from 0.013 to 0.149 (Table 5). The finding that the TNN5 and KHH2 populations, located in different cities, exhibited the lowest level of genetic differentiation was noteworthy. Based on the Mantel test for IBD, no significant correlation was found between genetic and geographical distances among captive populations (R^2^ = 0.0001, *p* = 0.469) (Figure 2).

**Figure 3:**
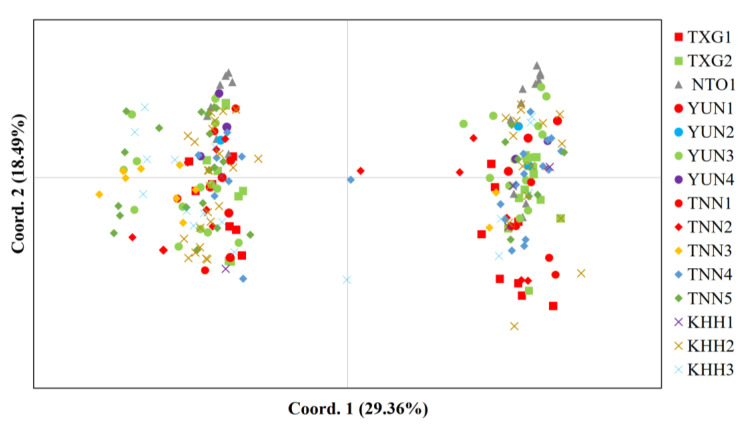
A two-dimensional plot of principal coordinate analysis (PCoA) of 239 individuals in 15 captive populations.

**Figure 4:**
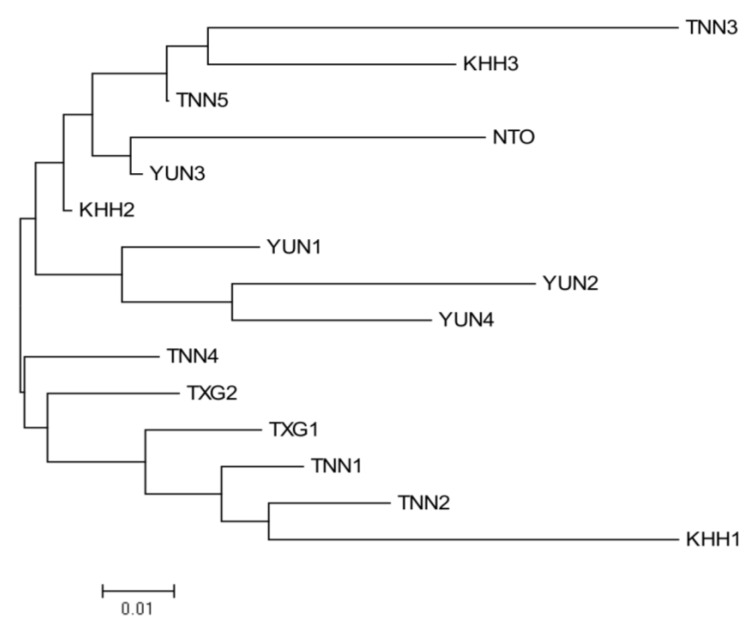
A neighbor-joining tree of *Rusa unicolor swinhoei* based on Nei’s genetic distance.

A principal coordinate analysis (PCoA) is also presented to summarize the individual relationships. The first component accounted for 29.36% of the total genetic variability, whereas the second and third components accounted for 18.49% and 14.60% of the variability, respectively (Figure 3). The PCoA biplot revealed the presence of two distinct genetic clusters, each comprising individuals from all captive populations. However, two individuals from the TNN2 and TNN4 populations could not be definitively assigned to either of the two clusters. The resulted neighbor-joining tree revealed a relatively similar result. Two main clusters were formed, with TXG1, TXG2, TNN1, TNN2, TNN4, and KHH1 populations in one cluster, whereas others joined together in a different cluster (Figure 4).

**Figure 5:**
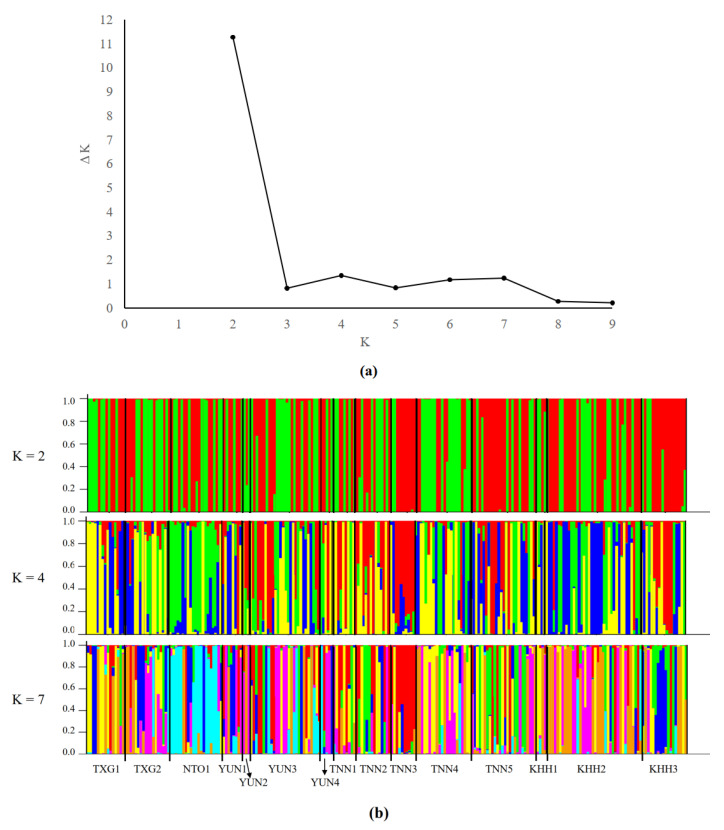
Barplots of genetic composition of individual for K = 2 and K = 4. (**a**) The scatter plots of ΔK; (**b**) Structure profile under K = 2, 4, and 7 with the highest ΔK value. Each individual is represented by a vertical bar, often partitioned into colored segments with the length of each segment representing the proportion of the individual’s genome. On the bottom of the plot, the name of population localities is indicated.

STRUCTURE clustering revealed a comparable genetic structure (Figure 5). Based on the ΔK values, a strong signal for K = 2 (ΔK = 11.274) and a comparatively weak signal for K = 4 (ΔK = 1.354) and K = 7 (ΔK = 1.242) were obtained (Figure 5a). Regardless of whether K = 2, 4, or 7, individuals from different captive populations were mixed with each other (Figure 5b). This implies that there was no clear population genetic structure among the captive populations, which is consistent with the results obtained from the PCoA (Figure 3) and Fst analysis (Table 5).

### 3.4. Parentage Analysis

The 13 microsatellites used in this study possessed high levels of polymorphisms and resolution, thus making them suitable for parental analysis. Maximum likelihood estimates from the ML-RELATE program were used for the 239 captive individuals. In total, 475 (1.670%) PO, 1035 (3.639%) FS, 3809 (13.394%) HS, and 23,122 (81.297%) U were identified among the 28, 441 pairs (2, 477 within-population pairs and 25, 964 inter-population pairs). Among the within-population pairs, we identified 81 (0.285%) PO, 222 (0.781%) FS, 425 (1.494%) HS and 1749 (6.150%) U relationships. For inter-population pairs, we found 394 (1.385%) PO, 813 (2.859%) FS, 3384 (11.899%) HS, and 21,373 (75.147%) U relationships. There were more inter-population pairs (4591 pairs,16.143%) with kinships than within-farm pairs (728 pairs, 2.560%) (Table 6).

## 4. Discussion

### 4.1. Resolution Power of Microsatellites for Individual Discrimination and Parentage Analysis

In Taiwan, the primary focus of deer farms is the extraction of deer velvet antlers, which is the main source of income. However, owing to the absence of pedigree records and the failure of farmers to follow scientific mating plans in their herds, there is a risk of inbreeding accumulation and a subsequent decrease in production potential within these farms. Microsatellites are powerful tools for paternity testing in the management of captive animal populations. This enabled accurate parentage determination and assisted in maintaining genetic diversity [44]. In this study, most of the microsatellite loci examined exhibited high levels of polymorphism. Botstein et al. [45] indicated markers with PIC values above 0.50 are considered to be highly informative, from 0.25 to 0.50 can be considered medially informative, and below 0.25 are essentially low informative. Four sets displayed low polymorphism (PIC < 0.25), four sets exhibited moderate polymorphism (0.25 < PIC < 0.50), and the remaining exhibited high polymorphism (PIC > 0.5) (Table 2). Among the microsatellite loci, Ca13 and Ca18 showed the highest allele numbers and discriminatory power. Notably, loci with the same allele number, such as Ca67, BM4107, RT1, and TGLA53 may display varying discriminatory powers. In contrast, we observed instances wherein loci with different allele numbers exhibited similar discriminatory power. Notably, Ca13 and Ca67 were two such loci that had comparable discrimination powers despite having different allele numbers (Table 2).

PD, PI, and PIsibs are the three essential parameters used in individual discrimination of parentage determination based on microsatellite data. The cumulative PD, PI, and PIsibs values based on all 13 polymorphic microsatellite loci were 0.999, 4.589 × 10^−7^, and 1.871 × 10^−3^, respectively (Figure 1). The cumulative PD, PI, and PIsibs values collectively demonstrated a high level of discrimination ability in individual identification and parentage determination. These parameters are essential in genetic studies and breeding programs, as they reflect the power of microsatellite markers to distinguish between individuals and accurately assign parentage. Additionally, parentage tests using microsatellites have become a valuable and widely adopted tool and gained significant importance in the breeding practices of various animals, including dogs [46], cats [47], horses [48], yak [49], and sika deer [50]. Previous studies recommended specific threshold values for PI (10^−3^ to 10^−4^) and PIsibs (<2 × 10^−2^) for wildlife forensic applications [51,52]. These microsatellite DNA markers could also be applied to future cases related to the poaching of wild Formosan sambar deer populations or other deer species.

### 4.2. Reduced Genetic Diversity in Captive Populations of Formosan Sambar Deer

Genetic diversity serves as the foundation for animal breeding because high genetic diversity is essential for meeting future human needs and preserving a rich gene pool, particularly in the face of climate change [12]. A high level of genetic diversity allows adaptation to changing climates and the development of breeds or strains suitable for specific environmental conditions [53]. However, market preferences tend to favor specific traits, disregarding the potential need for other varieties. This preference for a limited number of breeds leads to an increased probability of inbreeding and accumulation of harmful recessive alleles. Consequently, a decline occurs in the originally favorable traits, further contributing to the overall decrease in genetic diversity within a species [54]. As a result, artificially bred populations often experience genetic bottlenecks, leading to reduced genetic diversity when compared with wild populations [55].

The frequent inbreeding of individuals within a population can lead to decreased genetic diversity, resulting in reduced adaptability to the environment [13]. Allele number (A), observed heterozygosity (Ho), and expected heterozygosity (He) are indicators of genetic diversity. In the present study, we analyzed the genetic diversity of captive populations of Formosan sambar deer, with A = 3.092, Ho = 0.291, and He = 0.375 (Table 4). As expected, genetic variations in captive populations of Formosan sambar deer were lower than in wild populations of other deer, such as *C. elaphus montanus* in European (A = 7.944, Ho = 0.520, and He = 0.870) [56], *C. elaphus scoticus* in England (Ho = 0.506, and He = 0.801) [57], *C. nippon* in Japan (A = 5.000, Ho = 0.590, and He = 0.610) [58], and *C. nippon hortulorum* in China (A = 10.000, Ho = 0.849, and He = 0.787) [59]. Our investigation further revealed that the genetic diversity of Formosan sambar deer was lower than that of captive populations of *C. nippon pseudaxis* in Vietnam (A = 5.70, Ho = 0.574, and He = 0.600) [60], *Hydropotes inermis inermis* in China (A = 5.143, Ho = 0.531, and He = 0.662) [19], and *C. nippon* in Japan (A = 3.31, and Ho = 0.656) [20]. Additionally, when we compared our study to research involving both wild and captive sambar deer populations, the results consistently demonstrate a reduced level of genetic diversity in captive populations of Formosan sambar deer. For example, in comparison to the wild populations in the Western Himalayas (A = 12.60, Ho = 0.499, and He = 0.742) [61], and the captive populations in Taiwan (A = 13.50, Ho = 0.310, and He = 0.911) [27], our study consistently revealed lower levels of genetic diversity.

The reduced genetic diversity observed in the Formosan sambar deer may be attributed to inbreeding and the bottleneck effect, both of which can have substantial consequences on captive animal populations. In the present study, we observed that all captive populations displayed a deficiency in heterozygosity with positive F_IS_ values (Table 3). Furthermore, a significant bottleneck effect was detected in the entire captive population of Taiwan (*p* < 0.05 in SMM and TPM modes). These findings suggest that captive populations have experienced a reduction in genetic diversity and have undergone a substantial decrease in effective population size. The presence of a bottleneck effect indicates that the population has experienced a severe reduction in numbers, which can lead to the loss of genetic variation and increased vulnerability to genetic drift [62]. Anello et al. [63] conducted a genetic diversity analysis in captive populations (*Vicugna vicugna*) and found that translocation from the wild played a crucial role in effectively restoring genetic diversity in the captive populations. Our study highlights the importance of implementing effective genetic management strategies to prevent inbreeding and mitigate the effects of bottlenecks, thereby ensuring the long-term health and sustainability of captive populations of the Formosan sambar deer.

### 4.3. Low to Moderate Levels of Population Differentiation among Captive Populations

Population genetic structure, however, refers to the way genetic variation is distributed within and among populations. This structure is influenced by factors such as migration, genetic drift, and natural selection [64,65]. Understanding the population genetic structure helps in identifying distinct populations/germplasms within a species, which can be critical for effective management and conservation strategies. For instance, identifying genetically distinct populations/germplasms can help prevent inbreeding and promote gene flow between populations, thereby reducing the risk of genetic bottlenecks and improving overall genetic health.

The Formosan sambar deer has been an economically important animal in Taiwan since 1963. Unfortunately, the original sources of the captive populations remain unknown. Captive populations were divided into two major groups based on the PCoA (Figure 3). Similar results obtained by neighbor-joining tree (Figure 4) and the STRUCTURE analysis (Figure 5) revealed that the two separate gene pools probably represent the primary sources of captive populations of Formosan sambar deer. In a previous study, it was revealed that the Formosan sambar deer exhibited two distinct clades based on the D-loop region of the mitochondrial DNA, suggesting spatial divergence between the populations in North and South Taiwan [24]. Based on these findings, we proposed that the two genetic origins of the captive population probably originated from North and South Taiwan.

However, we also found that each genetic group comprised individuals from all captive populations (Figure 3 and Figure 5). These analyses demonstrate that frequent transfers occurred among captive populations of Formosan sambar deer in Taiwan. These results are consistent with the findings from the pairwise Fst analysis. According to Hartl and Clark [66], a commonly accepted scale is as follows: Fst < 0.05 indicates little genetic differentiation; Fst = 0.05–0.15 suggests moderate genetic differentiation; Fst = 0.15–0.25 indicates great genetic differentiation; Fst > 0.25 very great genetic differentiation. The majority of pairwise Fst values among all captive populations were below 0.05, with a few exceptions showing Fst values ranging from 0.05 to 0.15. This pattern suggested there is generally low to moderate levels of genetic differentiation among these captive populations (Fst = 0.013–0.149, as detailed in Table 5). Interestingly, captive populations located in different cities exhibited lower levels of genetic differentiation. This suggests that captive populations in various cities share genetic similarities. The captive population (TXG2) situated in central Taiwan and the population (KHH2) located in Southern Taiwan showed a low level of genetic differentiation (Fst = 0.023). Some populations located in adjacent cities showed moderate levels of genetic differentiation than those located in geographically distant cities. For example, TNN3 and KHH1, located in southern Taiwan, exhibited moderate levels of genetic differentiation (Fst = 0.140). A Mantel test of isolation by distance indicated no relationship between genetic distance and geographic distance among all captive populations, suggesting high translocation among captive populations in Taiwan (Figure 2).

In Taiwan, when a deer farm successfully breeds outstanding individuals with high velvet antler production, other farms may purchase their offspring or semen to improve herd productivity. Consequently, there may be frequent gene flow between different deer farms. According to the parentage analysis, there were more inter-population pairs (394 pairs, 1.385%) with parent–offspring kinships than within-farm kinships (81, 0.285%) (Table 4). Initially, frequent transfers among the captive populations led to an increase in antler production. However, over time, without a rigorous control strategy for mating, there is a possibility of inbreeding, resulting in a decline in antler production. Given that the current study did not include a comparison of the wild population of Formosan sambar deer, future investigations should focus on evaluating the genetic diversity and population genetic structure within the wild population to establish a basis for comparing disparities between the two groups.

## 5. Conclusions

The use of microsatellites in this study offers valuable insights into a wide range of ecological and evolutionary enquiries. Our findings indicated that captive populations of Formosan sambar deer exhibited lower levels of genetic diversity, which can be attributed to the effects of inbreeding and a bottleneck effect. To mitigate the adverse effects of inbreeding and to minimize the loss of genetic diversity, it is recommended to implement an action plan for captive populations that involves introducing new genetic material from wild populations. This study has the potential to enhance breeding management practices and aid in reducing the negative impacts of inbreeding on the production potential of Formosan sambar deer.

## Figures and Tables

**Table 1:** Localities, symbols, and sample numbers of 15 captive populations of *Rusa unicolor swinhoei* in Taiwan.

Symbols	City	Number
TXG	Taichung, Taiwan	
TXG1	Taichung, Taiwan	15
TXG2	Taichung, Taiwan	18
NTO	Nantou, Taiwan	
NTO1	Nantou, Taiwan	21
YUN	Yunlin, Taiwan	
YUN1	Yunlin, Taiwan	8
YUN2	Yunlin, Taiwan	3
YUN3	Yunlin, Taiwan	28
YUN4	Yunlin, Taiwan	5
TNN	Tainan, Taiwan	
TNN1	Tainan, Taiwan	9
TNN2	Tainan, Taiwan	14
TNN3	Tainan, Taiwan	10
TNN4	Tainan, Taiwan	22
TNN5	Tainan, Taiwan	26
KHH	Kaohsiung, Taiwan	
KHH1	Kaohsiung, Taiwan	4
KHH2	Kaohsiung, Taiwan	38
KHH3	Kaohsiung, Taiwan	18
Total		239

**Table 2:** Characterization of 13 microsatellite primer pairs used in this study.

Locus	Primer (5′-3′)	Repeat Motif	Tm (°C)	Reference
Ca13	F: CAGAAAGTTGTGAGGCACAG	(CA)_20_	60	[26]
	R: GTGGCCTCTGTTTCAGTGTA			
Ca18	F: TTCCGTCTCTCCCCTTAATA	(CA)_19_	56	[26]
	R: TGGATCTGAGATTTCTGCTG			
Ca30	F: CTATCCCATAGCCCAGTGAT	(GT)_15_	56	[26]
	R: TTTCCTCTTCCCTCTTCCTT			
Ca67	F: TAATCCTAACTCCTGGACCC	(GT)_16_	57	[26]
	R: CAAGAATTTTGGAGGGAAGC			
Ca71	F: TGCACACCCCCAGTCTGGT	(CT)_12_	60	[26]
	R: GTCTCACCTTTCCCATCAGC			
Cu02	F: GGGAGTCCTTCCTGTTCCTT	(CT)_9_	57	[27]
	R: CCAAGATCCCCCTTCTTGTT			
Cu05	F: AACAGCCTCACACACTCCAA	(AG)_9_	57	[27]
	R: CCTTTCTCTCTGTGGCCAGT			
Cu09	F: AGACATGCACAAGGCTCCTC	(AG)_10_	59	[27]
	R: GACTCCAAGCACTGGGATACA			
Cu10	F: CCCACTCGCACTCTCTCTCT	(AG)_18_	60	[27]
	R: ACTCAAGGGCCAGGGACTAT			
BM4107	F: AGCCCCTGCTATTGTGTGAG	(AC)_n_(TC)_n_(TG)_n_	55	[28]
	R: ATAGGCTTTGCATTGTTCAGG			
RT1	F: TGCCTTCTTTCATCCAACAA	(GT)_n_	56	[28]
	R: CATCTTCCCATCCTCTTTAC			
TGLA53	F: CAGCAGACAGCTGCAAGAGTTAGC	(AC)_n_	50	[28]
	R: CTTTCAGAAATAGTTTGCATTCATGCAG			
CEH-5	F: GAGCTGGTCCTCTGCGTGAT	(AC)_3_AA(AC)_11_	60	[29]
	R: CAGGCAGATTCTTTACCGTTG			

**Table 3:** Detailed genetic diversity parameters, discrimination power, and probability of identity of *Rusa unicolor swinhoei*.

Locus	A	Ho	He	F_IS_	PIC	HWE	PI	PIsibs	PD	Orders of PD
Ca13	14.000	0.561	0.727	0.228	0.695	*p* < 0. 05	0.107	0.413	0.727	2
Ca18	15.000	0.544	0.785	0.307	0.759	*p* < 0. 05	0.073	0.375	0.785	1
Cu02	2.000	0.009	0.009	−0.005	0.009	*p* > 0.05	0.982	0.991	0.009	12
Cu05	5.000	0.176	0.221	0.206	0.211	*p* < 0. 05	0.617	0.793	0.221	10
Ca67	7.000	0.706	0.731	0.034	0.683	*p* < 0. 05	0.121	0.415	0.731	3
Cu09	3.000	0.130	0.122	−0.067	0.115	*p* > 0.05	0.777	0.883	0.122	11
Cu10	6.000	0.374	0.346	−0.079	0.322	*p* < 0.05	0.452	0.690	0.346	9
Ca30	2.000	0.008	0.496	0.983	0.373	*p* < 0. 05	0.377	0.596	0.496	6
BM4107	7.000	0.431	0.452	0.047	0.415	*p* < 0. 05	0.337	0.608	0.452	7
RT1	7.000	0.452	0.628	0.281	0.569	*p* < 0. 05	0.198	0.485	0.628	4
Ca71	2.000	0.004	0.004	−0.002	0.004	*p* > 0.05	0.992	0.996	0.004	13
CEH-5	6.000	0.155	0.414	0.626	0.384	*p* < 0. 05	0.371	0.636	0.414	8
TGLA53	7.000	0.339	0.535	0.366	0.503	*p* < 0. 05	0.249	0.545	0.535	5
Average	6.385	0.299	0.421	-	0.388	-	-	-	0.421	-

A: number of alleles; Ho: observed heterozygosity; He: expected heterozygosity; F_IS_: inbreeding coefficient; PIC: polymorphism information content; HWE: Hardy–Weinberg equilibrium; PI: probability of identity for unrelated individuals; PIsibs: probability of identity for siblings; PD: power of discrimination.

**Table 4:** Statistics of genetic diversity of *Rusa unicolor swinhoei* in 15 captive populations in Taiwan.

Population	A	Ho	He	F_IS_
TXG	3.846	0.291	0.417	0.262
TXG1	2.769	0.241	0.374	0.268
TXG2	3.538	0.333	0.426	0.200
NTO				
NTO	2.231	0.256	0.299	0.146
YUN	4.231	0.311	0.404	0.212
YUN1	3.000	0.308	0.370	0.140
YUN2	2.231	0.308	0.346	0.054
YUN3	3.615	0.328	0.397	0.149
YUN4	2.462	0.231	0.355	0.305
TNN	5.385	0.312	0.424	0.250
TNN1	2.846	0.222	0.371	0.333
TNN2	3.308	0.346	0.424	0.185
TNN3	2.538	0.300	0.300	0.055
TNN4	3.923	0.322	0.398	0.191
TNN5	3.923	0.319	0.414	0.204
KHH	4.769	0.293	0.423	0.229
KHH1	2.385	0.250	0.317	0.176
KHH2	3.846	0.281	0.391	0.229
KHH3	3.769	0.326	0.449	0.245
Average	3.092	0.291	0.375	0.195

A, number of alleles; Ho, observed heterozygosity; He, expected heterozygosity; F_IS_, inbreeding coefficient.

**Table 5:** Pairwise genetic differentiation (below the diagonal) and *p*-values (above the diagonal) of *Rusa unicolor swinhoei* among 15 captive populations.

	TXG1	TXG2	NTO	YUN1	YUN2	YUN3	YUN4	TNN1	TNN2	TNN3	TNN4	TNN5	KHH1	KHH2	KHH3
TXG1	0.000	**0.028**	**0.001**	**0.159**	**0.004**	**0.019**	0.065	0.701	**0.061**	**0.002**	**0.003**	**0.007**	**0.002**	**0.027**	**0.009**
TXG2	0.032	0.000	**0.001**	0.209	**0.047**	**0.019**	0.093	0.165	**0.002**	**0.001**	**0.022**	**0.003**	**0.007**	**0.038**	**0.002**
NTO	0.097	0.065	0.000	**0.001**	**0.003**	**0.001**	**0.025**	**0.001**	**0.001**	**0.001**	**0.001**	**0.001**	**0.001**	**0.001**	**0.001**
YUN1	0.037	0.031	0.086	0.000	0.206	0.097	0.649	0.244	**0.007**	**0.001**	**0.042**	0.172	**0.003**	0.299	**0.023**
YUN2	0.093	0.053	0.092	0.058	0.000	**0.017**	0.494	**0.039**	**0.009**	**0.001**	**0.040**	**0.009**	**0.044**	**0.017**	**0.002**
YUN3	0.028	0.027	0.043	0.033	0.068	0.000	0.095	0.172	**0.006**	**0.001**	**0.008**	0.116	**0.002**	0.107	**0.002**
YUN4	0.081	0.062	0.086	0.048	0.054	0.063	0.000	0.218	**0.038**	**0.001**	0.124	0.256	**0.010**	0.277	**0.027**
TNN1	0.024	0.032	0.097	0.045	0.094	0.029	0.083	0.000	0.525	**0.005**	**0.018**	0.063	0.122	0.079	**0.004**
TNN2	0.043	0.043	0.095	0.063	0.081	0.040	0.082	0.031	0.000	**0.001**	**0.001**	**0.016**	**0.022**	**0.001**	**0.002**
TNN3	0.075	0.102	0.120	0.095	0.149	0.063	0.112	0.088	0.098	0.000	**0.001**	**0.002**	**0.001**	**0.002**	**0.001**
TNN4	0.037	0.032	0.070	0.039	0.057	0.032	0.060	0.052	0.060	0.092	0.000	**0.003**	**0.015**	0.061	**0.001**
TNN5	0.037	0.031	0.051	0.030	0.064	0.016	0.052	0.039	0.035	0.051	0.036	0.000	**0.001**	0.290	**0.003**
KHH1	0.073	0.060	0.120	0.108	0.096	0.071	0.139	0.062	0.063	0.140	0.058	0.079	0.000	**0.001**	**0.001**
KHH2	0.027	0.023	0.058	0.023	0.060	0.017	0.048	0.038	0.047	0.067	0.018	0.013	0.071	0.000	**0.005**
KHH3	0.043	0.039	0.077	0.046	0.084	0.036	0.074	0.058	0.046	0.069	0.062	0.030	0.106	0.035	0.000

Bold values are significantly different from 0 (*p* < 0.05).

**Table 6:** The kin relationships of *Rusa unicolor swinhoei* in 15 captive populations in Taiwan.

	PO	FS	HS	U
Within farms	81	222	425	1749
	(0.285%)	(0.780%)	(1.494%)	(6.149%)
Among farms	394	813	3384	21,373
	(1.385%)	(2.859%)	(11.899%)	(75.149%)
Total	475	1035	3809	23,122
	(1.670%)	(3.639%)	(13.393%)	(81.298%)

PO = Parent/Offspring, FS = Full Sibs, HS = Half Sibs, U = Unrelated.

## Data Availability

The data analyzed in this study are available from the corresponding author upon reasonable request.
